# Supplementary material for: Detection of SARS-CoV-2 in Saliva and Nasopharyngeal Swabs According to Viral Variants
Source: Microbiol Spectr. 2022 Nov 8;10(6):e02133-22. doi: 10.1128/spectrum.02133-22 (PMC9769595; doi:10.1128/spectrum.02133-22)
Supplement: Supplemental file 1 — Supplemental material. Download spectrum.02133-22-s0001.pdf, PDF file, 0.7 MB [file spectrum.02133-22-s0001.pdf]

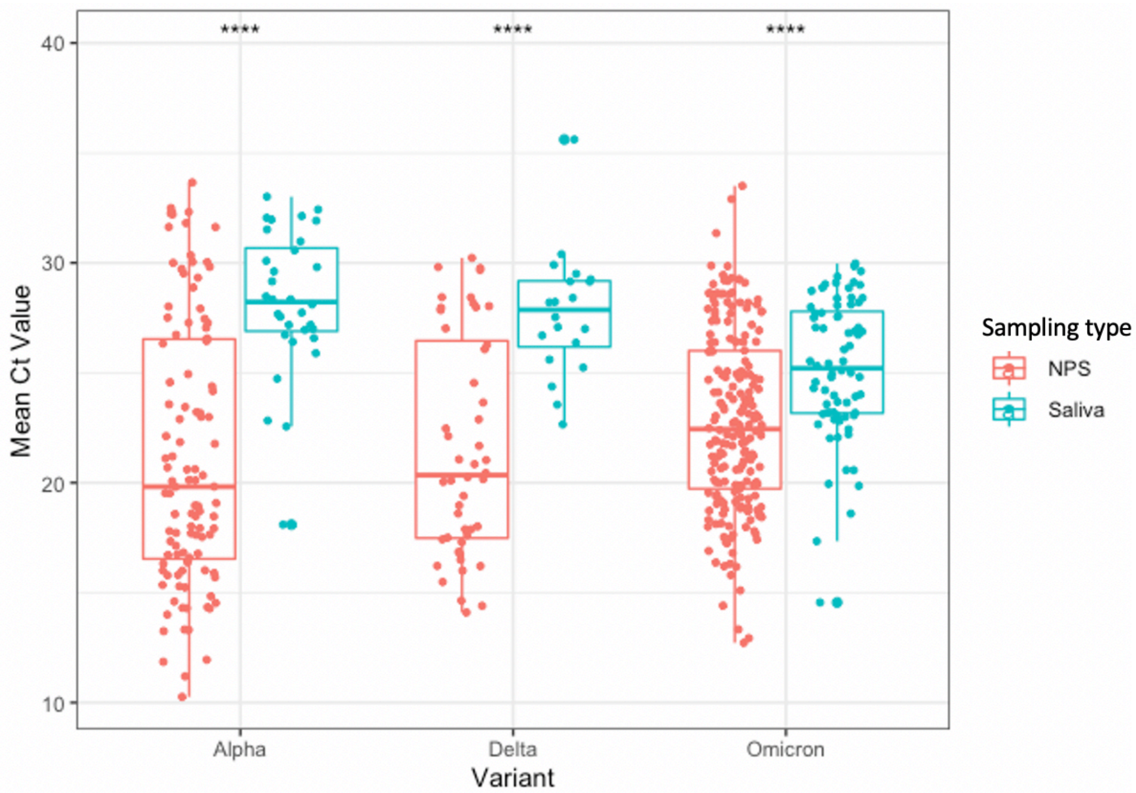

Supp Figure 1. SARS-CoV-2 Ct values for naso-pharyngeal swab (NPS) and saliva samples according to Alpha, Delta, and Omicron variants.

Boxes range from the first to third quartiles. Midlines represent median values. Individual points represent SARS-CoV-2–positive samples. Statistically significant differences ($p < 0.05$, ** $p<0.01$, *** $p<0.001$) between NPS and Saliva for each variant were determined by Mann–Whitney U-test or by Student's t-test.*

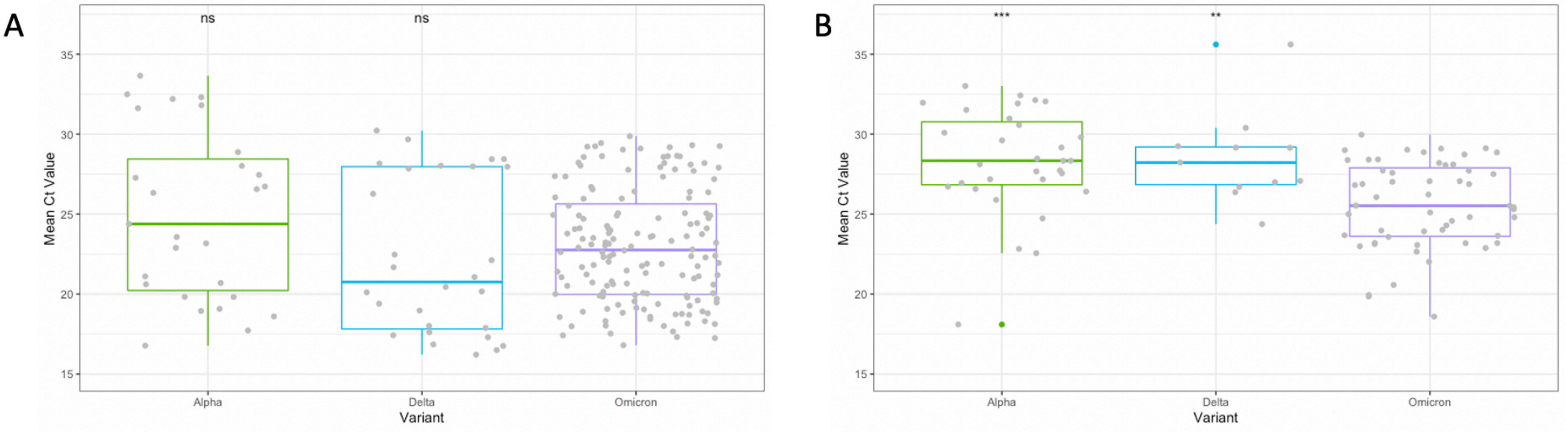

Supp Figure 2. Roche cobas® *SARS*-CoV-2 assay Ct values among Alpha, Delta, and Omicron variants in naso-pharyngeal swab (NPS) (A) and Saliva (B) samples.

*Boxes range from the first to third quartiles. Midlines represent median values. Individual points represent SARS-CoV-2–positive samples. Statistically significant differences (*p < 0.05, ** p<0.01,*** p<0.001) of Alpha and Delta versus Omicron were determined by Mann–Whitney U-test or by Student's t-test .*

GISAID virus name numbers of SARS-Cov2 whole genome sequences :

hCov-19/France/IDF-SLS-762111069335/2021 ; hCov-19/France/IDF-SLS-762111068761/2021 ; hCov-19/France/IDF-SLS-762111073787/2021 ;
hCov-19/France/IDF-SLS-762111077079/2021 ; hCov-19/France/IDF-SLS-762111083705/2021 ; hCov-19/France/IDF-SLS-762111080793/2021 ;
hCov-19/France/IDF-SLS-762111085044/2021 ; hCov-19/France/IDF-SLS-762111084651/2021 ; hCov-19/France/IDF-SLS-762111091673/2021 ;
hCov-19/France/IDF-SLS-762111093692/2021 ; hCov-19/France/IDF-SLS-762111092796/2021 ; hCov-19/France/IDF-SLS-762112003325/2021 ;
hCov-19/France/IDF-SLS-762112008470/2021 ; hCov-19/France/IDF-SLS-762112009743/2021 ; hCov-19/France/IDF-SLS-762112019105/2021 ;
hCov-19/France/IDF-SLS-762112018531/2021 ; hCov-19/France/IDF-SLS-762112018241/2021 ; hCov-19/France/IDF-SLS-762112028345/2021 ;
hCov-19/France/IDF-SLS-762112030150/2021 ; hCov-19/France/IDF-SLS-762112034673/2021 ; hCov-19/France/IDF-SLS-762112044785/2021 ;
hCov-19/France/IDF-SLS-762112042179/2021 ; hCov-19/France/IDF-SLS-762112047939/2021 ; hCov-19/France/IDF-SLS-762112047101/2021 ;
hCov-19/France/IDF-SLS-762112051058/2021 ; hCov-19/France/IDF-SLS-762112052614/2021 ; hCov-19/France/IDF-SLS-762112058059/2021 ;
hCov-19/France/IDF-SLS-762112061178/2021 ; hCov-19/France/IDF-SLS-762112061084/2021 ; hCov-19/France/IDF-SLS-762112064050/2021 ;
hCov-19/France/IDF-SLS-762112065420/2021 ; hCov-19/France/IDF-SLS-762112065423/2021 ; hCov-19/France/IDF-SLS-762112071071/2021 ;
hCov-19/France/IDF-SLS-762112066878/2021 ; hCov-19/France/IDF-SLS-762112067406/2021 ; hCov-19/France/IDF-SLS-762112067482/2021 ;
hCov-19/France/IDF-SLS-762112068811/2021 ; hCov-19/France/IDF-SLS-762112068868/2021 ; hCov-19/France/IDF-SLS-762112068912/2021 ;
hCov-19/France/IDF-SLS-762112069011/2021 ; hCov-19/France/IDF-SLS-762112069650/2021 ; hCov-19/France/IDF-SLS-762112071127/2021 ;
hCov-19/France/IDF-SLS-762112071771/2021 ; hCov-19/France/IDF-SLS-762112072654/2021 ; hCov-19/France/IDF-SLS-762112073611/2021 ;
hCov-19/France/IDF-SLS-762112076896/2021 ; hCov-19/France/IDF-SLS-762112075551/2021 ; hCov-19/France/IDF-SLS-762112076117/2021 ;
hCov-19/France/IDF-SLS-762112076127/2021 ; hCov-19/France/IDF-SLS-762112076189/2021 ; hCov-19/France/IDF-SLS-762112076940/2021 ;
hCov-19/France/IDF-SLS-762112077426/2021 ; hCov-19/France/IDF-SLS-762112079789/2021 ; hCov-19/France/IDF-SLS-762112079805/2021 ;
hCov-19/France/IDF-SLS-762112079816/2021 ; hCov-19/France/IDF-SLS-762112089662/2021 ; hCov-19/France/IDF-SLS-762112090173/2021 ;
hCov-19/France/IDF-SLS-762112089531/2021 ; hCov-19/France/IDF-SLS-762112089650/2021 ; hCov-19/France/IDF-SLS-762112089699/2021 ;
hCov-19/France/IDF-SLS-762112090264/2021 ; hCov-19/France/IDF-SLS-762112090514/2021 ; hCov-19/France/IDF-SLS-762112090527/2021 ;
hCov-19/France/IDF-SLS-762112090974/2021 ; hCov-19/France/IDF-SLS-762201003960/2022 ; hCov-19/France/IDF-SLS-762201003652/2022 ;
hCov-19/France/IDF-SLS-762201030716/2022 ; hCov-19/France/IDF-SLS-762201030963/2022 ; hCov-19/France/IDF-SLS-762201031748/2022 ;
hCov-19/France/IDF-SLS-762202003824/2022 ; hCov-19/France/IDF-SLS-762202007355/2022 ; hCov-19/France/IDF-SLS-762101101664/2021 ;
hCov-19/France/IDF-SLS-762101101751/2021 ; hCov-19/France/IDF-SLS-762102003663/2021 ; hCov-19/France/IDF-SLS-762102003101/2021 ;
hCov-19/France/IDF-SLS-762102011623/2021 ; hCov-19/France/IDF-SLS-762102017734/2021 ; hCov-19/France/IDF-SLS-762102017876/2021 ;
hCov-19/France/IDF-SLS-762102015848/2021 ; hCov-19/France/IDF-SLS-762102018004/2021 ; hCov-19/France/IDF-SLS-762102023608/2021 ;
hCov-19/France/IDF-SLS-762102024194/2021 ; hCov-19/France/IDF-SLS-762102029131/2021 ; hCov-19/France/IDF-SLS-762102032207/2021 ;
hCov-19/France/IDF-SLS-762102037054/2021 ; hCov-19/France/IDF-SLS-762102041403/2021 ; hCov-19/France/IDF-SLS-762102047074/2021 ;
hCov-19/France/IDF-SLS-762102048430/2021 ; hCov-19/France/IDF-SLS-762102048996/2021 ; hCov-19/France/IDF-SLS-762102052308/2021 ;
hCov-19/France/IDF-SLS-762102055793/2021 ; hCov-19/France/IDF-SLS-762102058821/2021 ; hCov-19/France/IDF-SLS-762102053035/2021 ;
hCov-19/France/IDF-SLS-762102056673/2021 ; hCov-19/France/IDF-SLS-762102059157/2021 ; hCov-19/France/IDF-SLS-762102058833/2021 ;
hCov-19/France/IDF-SLS-762102063781/2021 ; hCov-19/France/IDF-SLS-762102061345/2021 ; hCov-19/France/IDF-SLS-762102070212/2021 ;
hCov-19/France/IDF-SLS-762102072023/2021 ; hCov-19/France/IDF-SLS-762102077510/2021 ; hCov-19/France/IDF-SLS-762102082352/2021 ;
hCov-19/France/IDF-SLS-762102083256/2021 ; hCov-19/France/IDF-SLS-762102083264/2021 ; hCov-19/France/IDF-SLS-762103003119/2021 ;

hCov-19/France/IDF-SLS-762103013192/2021 ; hCov-19/France/IDF-SLS-762103059603/2021 ; hCov-19/France/IDF-SLS-762103080753/2021 ; hCov-19/France/IDF-SLS-762103085636/2021 ; hCov-19/France/IDF-SLS-762103088014/2021 ; hCov-19/France/IDF-SLS-762103090904/2021 ; hCov-19/France/IDF-SLS-762103091529/2021 ; hCov-19/France/IDF-SLS-762103101811/2021 ; hCov-19/France/IDF-SLS-762104002994/2021 ; hCov-19/France/IDF-SLS-762104038729/2021 ; hCov-19/France/IDF-SLS-762104043278/2021 ; hCov-19/France/IDF-SLS-762104052661/2021 ; hCov-19/France/IDF-SLS-762104064987/2021 ; hCov-19/France/IDF-SLS-762104097047/2021 ; hCov-19/France/IDF-SLS-762104098612/2021 ; hCov-19/France/IDF-SLS-762105010666/2021 ; hCov-19/France/IDF-SLS-762105024259/2021 ; hCov-19/France/IDF-SLS-762105080592/2021 ; hCov-19/France/IDF-SLS-762107057879/2021 ; hCov-19/France/IDF-SLS-762107072141/2021 ; hCov-19/France/IDF-SLS-762108070774/2021 ; hCov-19/France/IDF-SLS-762110087396/2021 ; hCov-19/France/IDF-SLS-762110097890/2021 ; hCov-19/France/IDF-SLS-762111009230/2021 ; hCov-19/France/IDF-SLS-762111027078/2021 ; hCov-19/France/IDF-SLS-762111036734/2021 ; hCov-19/France/IDF-SLS-762111040555/2021 ; hCov-19/France/IDF-SLS-762111048861/2021 ; hCov-19/France/IDF-SLS-762111067035/2021
